# Flash Flood Vulnerability Mapping Based on FFPI Using GIS Spatial Analysis Case Study: Valea Rea Catchment Area, Romania

**DOI:** 10.3390/s22093573

**Published:** 2022-05-07

**Authors:** István Kocsis, Ștefan Bilașco, Ioan-Aurel Irimuș, Vasile Dohotar, Raularian Rusu, Sanda Roșca

**Affiliations:** 1 Faculty of Geography, Babes-Bolyai University, 400006 Cluj-Napoca, Romania; kfistvan@yahoo.com (I.K.); ioan.irimus@ubbcluj.ro (I.-A.I.); vasile.dohotar@ubbcluj.ro (V.D.); raularian.rusu@ubbcluj.ro (R.R.); sanda.rosca@ubbcluj.ro (S.R.); 2 Cluj-Napoca Subsidiary Geography Section, Romanian Academy, 400015 Cluj-Napoca, Romania

**Keywords:** flash flood, spatial analysis, GIS model, weight of evidence, FFPI

## Abstract

The risk associated with extreme hydrological processes (flash floods, floods) is more present than ever, taking into account the global climatic changes, the expansion of inhabited areas and the changes emerging as a result of inadequate land management. Of all the hydrological risks, slope flash floods represent the processes that have the highest impact because of the high speed of their development and their place of origin, which makes them difficult to predict. This study is performed in an area susceptible to the emergence of slope flash floods, the Valea Rea catchment area, spatially located in Northwest Romania, and exposed to western circulation, which favours the development of such processes. The entire research is based on a methodology involving the integration of spatial databases, which indicate the vulnerability of the territory in the form of a weighted average equation to highlight the major impact of the most relevant factor. A number of 15 factors have been used in raster spatial databases, obtained by conversion (land use, soil type, lithology, Hydrologic Soil Group, etc.), derived from the digital elevation model (slope, aspect, TWI, etc.) or by performing spatial analysis submodels (precipitation, slope length, etc). The integration of these databases by means of the spatial analysis equation based on the weighted average led to the vulnerability of the territory to FFPI, classified on five classes from very low to very high. The final result underlines the high and very high vulnerability (43%) of the analysed territory that may have a major impact on the human communities and the territorial infrastructure. The results obtained highlight the torrential nature of the analysed catchment area, identifying several hotspots of great risk, located mainly within the built-up areas of intensely inhabited regions; a fact which involves a major risk and significant potential material damage in the territory. The model was validated by directly comparing the results obtained with locations previously affected, where the flood effects have been identified, highlighting the fact that the model may be taken into account to be applied in practice, and also to be implemented in territories that share the same features.

## 1. Introduction

As a consequence of the climate changes in recent decades, the frequency, magnitude, and duration of several weather and climate-related extreme events have been modified at the global, European and regional level [1]. There are no doubts regarding the fact that all these changes may be the result of the increasing complexity of anthropogenic activities. In the European Environment Agency report [1], it is shown that even if the emissions of CO_2_ and other greenhouse gases are reduced to zero in the near future, the time of atmospheric residence of the greenhouse gases, as well as the dynamic of the climate system, would still continue to generate anthropogenic climate changes for several decades by increasing record-high temperatures, the rate of extreme precipitation events and drought periods, and leading to more frequent and longer heat waves.

Related to these atmospheric extremes, the frequency of destructive events, such as flash floods, is also increasing. Flash floods are generated by intense local precipitation events and represent one of the most dangerous and unpredictable natural disasters. They occur so quickly that people are caught off-guard [2]. Together with river floods and storms, they are the most important natural hazard in Europe in terms of economic losses, and as a result of damage to infrastructure, property, and agricultural land, they are responsible for almost one-third of the damage caused by all natural hazards [1].

Every year, flooding, in general, is responsible for more deaths around the world than any other type of natural disaster. The statistics revealed the fact that countries from Eastern and Southern Europe, more precisely Romania, Bulgaria, Slovakia, and Serbia, have the highest deaths per million inhabitants (>10) related to flooding for the period from 1991–2015. According to EEA 2017 [1], river floods are estimated to affect approximately 300,000 people per year in the EU by the 2050s and 390,000 people by the 2080s.

As a result, integrative flood-risk management is a vital issue [3]. For sustainable disaster and flood-risk management, international cooperation is required, as well as a disaster statistic database, which includes the disaster occurrences, their effect upon people, and the economic losses to regions and countries [4]. Since 1988, the Centre for Research on the Epidemiology of Disasters (CRED) has implemented and maintained an Emergency Events Database, which includes disaster events that occurred in Romania too (the flood in 2015, the storm in 2017, severe weather in 2019, and floods in 2020). There are many disaster databases at the national level or that are related to a specific type of hazard: SIGMA disaster-event database maintained by SwissRe; DisDAT, initiated by the Global Risk Identification Program (GRIP) and CRED, which brings together publicly available disaster-related data from different countries (GRIp, 2010) [4]; Documentation of Mountains Disasters—DOMODIS (1998–2002). 

The European Flood Directive 2007/60/CE of the European Parliament and the Council was developed for flood risk assessment and management upon European rivers. To this end, the member states must identify river basins at risk of flooding, draw hazard and flood risk maps (such as Elba Flood Atlas, Danube Atlas—hazard and risk maps, 2012), and set up flood risk management plans for those areas [5]. Flash flood events all over Europe were also analysed through numerous international research projects between different institutions, such as the HYDRATE Project, Integrated flood risk analysis and management methodologies—FLOODsite [6], European flood forecasting system—EFFS [7].

Probably due to the better disaster management and warning systems, the number of human fatalities in relation to natural disasters shows a decreasing trend, but the number of people affected follows the increasing trend of the number of events [4].

As a result of selecting and summarising the most cited scientific papers written between 1990 and 2018 concerning vulnerability to floods and susceptibility assessment, Rehman (2018) revealed that most of the research approached flash floods, urban floods, and coastal floods. Given the catastrophic consequences inflicted by these devastating phenomena, and the capacity of the authorities to manage emergency and recovery situations, flash floods should be studied as a matter of priority in developing countries [4,8]. 

Therefore, the identification of areas susceptible to flash floods within catchment areas represents a paramount measure in the prevention, control, and mitigation of the catastrophic effects on human society [9,10]. Based on the scientific literature, two types of flood analysis methods can be distinguished, deterministic modelling and parametric methods. Because they use easily accessible, site-specific information to build up an image about the areas prone to natural disasters, parametric methods became widely applied.

To help the conventional prediction methods, Flash Flood Monitoring and Prediction System (FFMS) [11], for example, were set up as methods to identify the areas susceptible to flash floods. By overlaying only four geographical variables (slope, vegetation, soil type, and land use) in the GIS environment, was determined for the first time the basics of the Flash Flood Potential Index (FFPI) within the National Weather Service, “Western Region Flash Flood Project” (USA). After his method was successfully tested during flash flood situations in several geographical regions, such as Colorado, central New York, Northeast Pennsylvania and central Iowa, many researchers manifested their interest in modifying the original formula. Changes related to weighting factors [12], as all elements received equal weighting [13], while additional weighting was given to Slope and Land Use/Cover [14], and later a new factor was added, the annual maximum daily rainfall statistics [15].

The methodology was gradually improved by taking into account a higher number of geographical variables and weighting these factors into the final equation of FFPI. In the last decades, several methods were developed for natural hazards, especially for landslide and flood vulnerability mapping.

Recent methods such as geospatial techniques [16,17,18,19,20], bivariate statistical methods, frequency ratio (FR) [21], weights-of-evidence (WofE) [22,23,24], machine learning techniques [25,26], hybrid computational approaches [27,28], and decision tree [29] were proposed in the literature.

In Romania, due to the morphological conditions given by the presence of the Carpathians, the drawing up of studies determining the FFPI for several regions became a necessity. These studies were based on the above-mentioned methods and were performed by many authors, such as [30,31,32,33,34,35,36].

The FR and WofE are the most popular bivariate statistical analysis (BSA) techniques used for flash flood susceptibility mapping. The basic feature of the BSA is that the events are conditioned by the same combination of factors throughout the entire study area [37]. WofE can be applied considering even several predictive variables [38,39,40,41].

The effects inflicted by flash floods in the analysed area have a very high impact on the infrastructure and the communities in the Valea Rea catchment, and they are visible after each torrential rain of heavy intensity. The policy to reduce the management costs for small catchment areas involved a lack of their monitoring. This makes it difficult to directly identify the associated risk. Therefore, the development of spatial analysis models by integrating the factors which contribute to the identification of hotspots affected by flash floods is a useful method and it is compulsory to take it into account within the studies of territorial technical assessment. The above-mentioned references clearly highlight the usefulness and the necessity of implementing of such models. The need for the current paper is pressing and dire because of the absence of hydrometric data in the study area, correlated with a total lack of interest from the side of the authorities concerning the regionalisation of flash flood risks to mitigate their effects.

The purpose of this current research study is to assess the flood susceptibility map applying the WofE bivariate statistical GIS-based approach model in the Valea Rea catchment area, Romania.

## 2. Study Area

**Figure 1:**
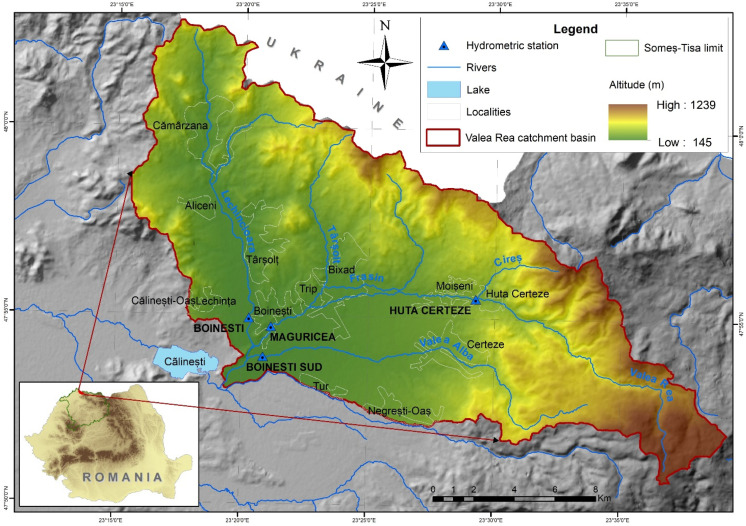
Valea Rea River catchment area location.

The study area corresponds to the Valea Rea River catchment, located in the Northwest part of Romania (Figure 1) in the Maramureș Carpathian region, covering an area of 293 km^2^. The Valea Rea River (which would translate as “Bad Valley”) is a right tributary of the Tur River, upstream of the Călinești-Oaș Reservoir. Draining the Oaș and Gutâi Mountains and Oaș Basin, the catchment elevation ranges from 145 to 1239 m asl. With an average altitude of 410 m asl., the average slope of 10.10, and the circularity ratio [41] of 0.33, the accelerated surface runoff and flash flood occurrence conditions are given [42].

In an ample analysis regarding the flash floods produced within the last 30–40 years, it is shown that the study area is part of the category of most vulnerable regions within the Someș-Tisa drainage space, having a high potential for the occurrence of flash flood phenomena [43]. Generally, the entire area of the Valea Rea catchment is affected by a high number of flash floods, and these events occur throughout the entire year.

The average multiannual discharge of Valea Rea at the Huta Certeze station is 1.83 m^3^/s (for the period from 1966–2012). During the most severe flash floods, the discharge reached peaks, for example, of: 89.6 m^3^/s in May 1970, 47.2 m^3^/s in June 1974, 48.2 m^3^/s in December 1979, 55.9 m^3^/s in December 1993, 62.0 m^3^/s in June 2013, 42.3 m^3^/s in June 2018, 46.6 m^3^/s in August 2019.

As a consequence, flash floods and torrential runoff extreme phenomena affect the lives of the inhabitants and social-economic material assets in the entire study area, in rural settlements, such as Huta Certeze, Certeze, Moișeni, Bixad, Trip, Boinești, Lechința, Târșolț, Aliceni, and Cămârzana. Among these, Cămârzana is on the select list of settlements which were predicted to suffer the highest potential damage between 2014 and 2020, including 219 households, 519 ha of land, 3.9 km of county roads, and 7 km of national roads. Boinești (more than 23 flash floods) and Huta Certeze (more than 15 flash floods) are among the hydrometric stations which recorded the highest number of flash floods during the entire period of their existence [44].

Representing an area that has a high potential for the occurrence of flash floods, the Valea Rea catchment area was also analysed in other scientific works [36].

## 3. Methodology and Database

The high complexity of the spatial analysis model presented in this research supposes the approach of a methodology that allows for the management of spatial and alphanumeric databases in such a way as to highlight the local and general specificity of each database which makes up the final model. The analysis of each component and their integration in the form of spatial analysis equations based on mathematical equations and weighted averages finalises the model and allows the spatial identification of areas that have different types of vulnerabilities to flash floods.

**Figure 2:**
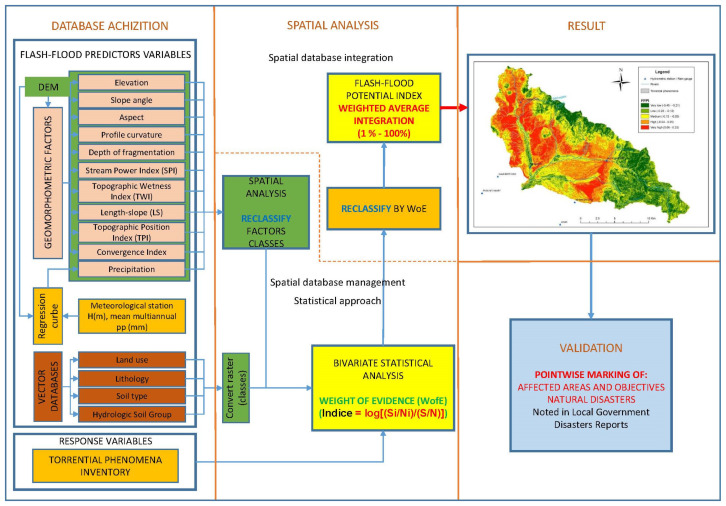
Flowchart of the methodology.

The proposed methodology is structured on four main stages, starting from the database acquisition, then the performance of the detailed spatial analysis, the presentation of the final results, and the validation of the results for the proposed model in order to be scientifically applied in practice (Figure 2). There is an integrated approach of the methodological stages, both vertically and horizontally, in order to have proper management of the many (raster, vector, alphanumeric data) types of spatial databases.

The basic stage for the development of the proposed model is represented by the database acquisition stage, which fundaments the proposed model. In this stage, the direct acquisition of databases is performed by downloading (DEM obtained on the basis of LIDAR flights with a spatial resolution of 3 m, CLC), vectorization (lithology, soils, and spatial extension of the existing torrents), and tabular data acquisition (coordinates of meteorological and pluviometric stations, multiannual average amount of precipitation).

Another manner of database acquisition refers to indirect acquisition by deriving the spatial databases according to the functions made available by specialised software. Such an acquisition was performed for the spatial identification of the slope angle value, the slope aspect, the plan curvature, HSG, TPI, SPI, and TWI. These databases were derived from the DEM and were integrated in the form of raster databases for the stage of spatial analysis.

The high complexity of the spatial databases which make up the proposed model determines the identification of two distinctive substages within the stage of database acquisition. Therefore, there is one substage of spatial analysis which integrates the derived databases and those collected by direct acquisition to obtain final results, outlined as entry databases in the proposed model (L-S, Depth of fragmentation). The other substage integrates a distinctive spatial analysis submodel to spatialise the multiannual average amount of precipitation taking into account the spatial location of the meteorological and pluviometric stations and performing a statistical correlation between altitude and the average amounts of precipitation.

The development of such a submodel increases the complexity of the main model, resulting in the management of database acquisition to pass from the stage of simple acquisition to the stage of integrated management based on the efficient use of all resources related to available spatial and non-spatial databases to finalise the model. The spatial analysis submodel proposed for the spatialisation of the average amount of precipitation according to altitude is based on the statistical spatial analysis implemented according to spatial analysis equations obtained as a result of the identification of the regression line and its equation. The regression line best approximates the correlation of the average precipitation values taking into consideration the natural evolution of the modelled phenomenon. Therefore, the following equation has been used for the entire study area:

Y = a + b ln(x)

where

Y = average precipitation, a = −148.377, b = 190.707, x = altitude

The equation was obtained by implementing the precipitation and altitude values for a number of nine stations unevenly distributed across the study area and its immediate vicinity, by means of Curveexpert software. A correlation coefficient of 0.980 was obtained, providing a 95% confidence coefficient.

The spatial analysis stage is the main and fundamental stage for finalising the proposed model to identify the vulnerable areas. It spatially and structurally integrates the databases that enter the model to provide the final result. The development of the main stage of spatial analysis is based on the implementation of two spatial analysis submodels that are different in terms of manner of implementation. They are developed according to spatial analysis equations derived from different equations in terms of structuring manner, as one model is based on a bivariate statistical equation and the other model is based on a deterministic equation that integrate the spatial databases resulting from the implementation of the first model. The model based on statistical analysis is centered on the bivariate equation WofE and allows the analysis of the basic components of the model to identify the behaviour of each analysed factor concerning the statistical answer to flash flood occurrence. The statistical equation used for the implementation of the model and the identification of the numerical value of each interval for every analysed parameter was developed by [45,46,47] in the form of:

Index = log[(Si/Ni)/(S/N)](1)

where: 

Index = the statistical value of the interval within the analysed factor;

Si = the area (sq km) with torrents on an interval of the analysed factor;

Ni = the total area covered by the analysed factor (sq km);

S = the total area with torrents within the entire study area (sq km);

N = the total area with torrents within the entire study area (sq km).

The finalisation of the spatial analysis submodel involves several main substages that are highly correlated to each other and converge to the acquisition of numeric databases which are integrated in a statistical formula, highlighting the statistical behaviour of each interval of the analysed factor. One main stage is represented by the uniformisation of the types of databases that enter the spatial analysis model, taking into account that different types of databases, such as raster and vector databases, were acquired during the stage of database acquisition. Taking into consideration the fact that the entire spatial analysis model is based on the implementation of spatial analysis equations on raster structures, it was decided that the vector format databases representing CLC, lithology, soil type, and HSG be converted to raster format according to the class used in the analysis. The resolution of the raster databases acquired as a result of conversion is three, which is equal to the one of the spatial databases derived from DEM.

The second stage of the submodel is represented by the spatial integration of the analysed factors with the areas covered by torrents in order to extract numerical values for areas, to be introduced in the statistical equation for computing. This stage has in its centre the overlay vector–raster analysis, integrating on one hand the vector databases representing the spatial extension of the torrents, and on the other hand the raster databases representing each factor classified according to its susceptibility to flash floods.

The second main submodel of the spatial analysis stage proposes the integration of all the factors unitarily analysed within the statistical submodel based on the WofE equation according to a deterministic spatial analysis equation of the weighted average type.

The integration of the two submodels in the spatial analysis stage is made according to the reclassification method, the main purpose of which is the acquisition of digital data in raster format to highlight spatially the numeric values of every factor and its degree of susceptibility to flash floods and to allow their integration on the basis of the deterministic equation. The deterministic equation was implemented in the GIS environment in the following form:

(“BSA_SPI.tif” × 2) + (“BSA_LS.tif” × 8) + (“BSA_PP.tif” × 7) + (“BSA_DepFrag_ha.tif” × 8) + (“BSA_TPIndex3.tif” × 5) + (“BSA_DEM.tif” × 2) + (“BSA_Convergente_Index.tif” × 7) + (“BSA_Profile_curv.tif” × 8) + (“BSA_Aspect.tif” × 3) + (“BSA_Slope.tif” × 15) + (“BSA_HSG_cor.tif” × 10) + (“BSA_Lithology_cor.tif” × 2) + (“BSA_CLC.tif” × 10) + (“BSA_SOL_Tip.tif” × 5) + (“BSA_TWI.tif” × 8)/15(2)

where: “BSA_SPI.tif” = analysed factor, 2 = percentage weighting the factor, +/× = mathematical identifiers.

The specific weight of each factor was established according to the importance and influence of the factor within the general process related to the emergence and development of flash floods.

The third stage represents the acquisition and analysis of the spatial impact of the final result. To perform the spatial analysis of the impact, the final result was classified on five FFPI vulnerability classes, from low vulnerability to very high vulnerability. Each class was identified numerically in intervals based on the Natural Breaks classification implemented in ArcMap 10.7.1 software.

The validation stage represents the last stage in the logical succession of the proposed spatial analysis model, and it is absolutely necessary to be carried out so that the obtained results may be scientifically implemented in practice and the model be implemented in other territories that share similar features. Therefore, the model validation is based on the method of direct comparison between the results obtained as a result of running the proposed model and the reality in the field. In this case, several locations within the study area have been selected, as they were identified in the synthetic reports performed when natural calamities were recorded. They were analysed according to the impact identified on the basis of the model. The model will be considered valid if the high and very high vulnerability classes have a direct impact on the identified locations.

The development of spatial analysis models in the form of whitebox-type methodology based on stages, substages, and integration between them leads to better database management and provides the opportunity for the automation of the work flows if necessary.

## 4. Results

The results obtained as a consequence of the implementation of the methodology materialised in spatial analysis models and submodels are first of all analysed individually, both from the perspective of the specific database acquisition for every factor taken into account, and from the perspective of the territorial impact of the analysed factor. Secondly, the analysis of the obtained results is performed by correlating the involved factors in the general model of spatial analysis to obtain the territorial specificity leading to different intensities of the FFPI.

The territorial specificity from the point of view of the FFPI is given by the statistically-based integrated analysis of a number of 15 factors which best highlight the territorial development of the analysed process.

### 4.1. Elevation

**Figure 3:**
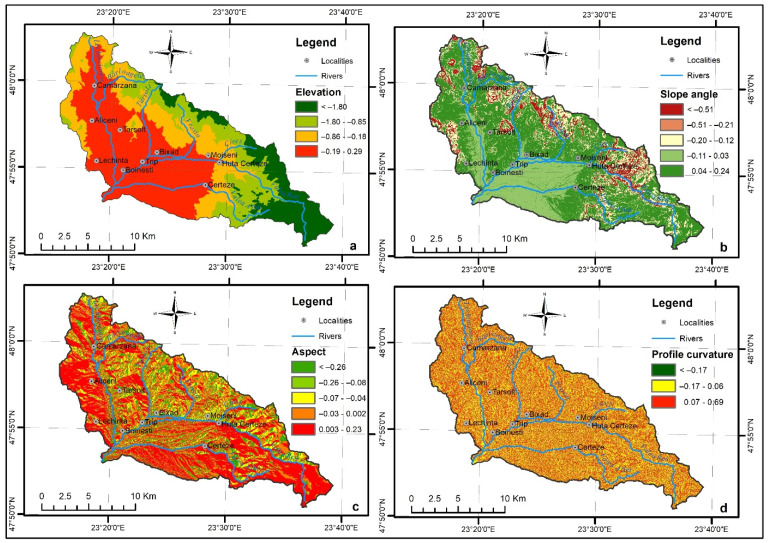
(**a**) Elevation, (**b**) Slope angle, (**c**) Aspect, (**d**) Profile curvature.

Elevation is one of the most decisive factors in terms of climate features, as it influences the vegetation zoning, the precipitation pattern, and, implicitly, the formation of the runoff conditions. The altitude map of the study area was derived from DEM into five classes, and to highlight the morphological levels we used the manual classification method: 145–300, 300–450, 450–650, 650–850, 850–1239 m (Figure 3a). The highest value of the final weight (WofE) was 0.29, corresponding to the 145–300 m interval, emphasising the fact that floods are concentrated on the lower sector of the drainage basin. The highest morphological level between 850 and 1239 m does not have any torrential formations, so its importance in computing the WofE is zero. Its Weighted Average Integration (WAI) percent in the FFPI map is 2% (Table 1).

### 4.2. The Slope Angle

The slope angle (Figure 3b) represents one of the most basic physiographic conditions in the formation of surface runoff processes, directly influencing the infiltration and the velocity of the water flow. As the slope angle increases, the drainage process, the sediment transport capacity, and, implicitly, the erosivity are accentuated. The Slope tool in the Spatial Analyst extension of ArcMap 10.7.1 software was used in order to obtain the spatial distribution of slope angle values. The following five classes were selected according to the literature [26]: <30, 3.1–70, 7.1–150, 15.1–250, >250. Positive WofE values were assigned to intervals 3.1–70 (0.24) and 7.1–150 (0.04), which include all the existing torrential formations (Table 1). As well as the LULC, it received the highest WAI percent in the FFPI computation (15%).

### 4.3. The Slope Aspect

The slope aspect (Figure 3c) has an indirect impact on hydrological processes due to its influence on evapotranspiration, soil humidity, and the direction of frontal precipitation [46]. With the use of the Aspect tool from the Spatial Analyst extension of ArcMap 10.7.1 software, these flash flood predictor values were divided into five slope aspect classes, of which most face Southeast/West (28.7%) and East/Northeast (24.1%). Areas affected by torrentiality are located on slopes that have a North/Northeast aspect, representing 29.3% of the torrential pixels, and received the highest value of the WofE, 0.23. For the final FFPI map computation, it counted 3% (Table 1).

### 4.4. The Profile Curvature

The profile curvature (Figure 3d) represents a flood conditioning variable used to compute the FFPI and indicates the direction of maximum slope. The values of this variable can be used to distinguish between areas with accelerated runoff and those with decelerated runoff. The negative value indicates that the surface is upwardly convex at the cell and flow will be decelerated, while the positive value indicates that the surface is upwardly concave at that cell and the flow will be accelerated. The profile curvature was extracted from DEM, applying the Curvature tool from the Spatial Analyst extension of ArcMap 10.7.1 software. The manual classification method was used to categorize its values into three classes: (−209)–0, 0.1–1.92, 1.9–199. As was expected, concave slopes received the highest WofE value (0.69). In the final FFPI equation, it received a weight of 8% (Table 1).

### 4.5. The Depth of Fragmentation

**Figure 4:**
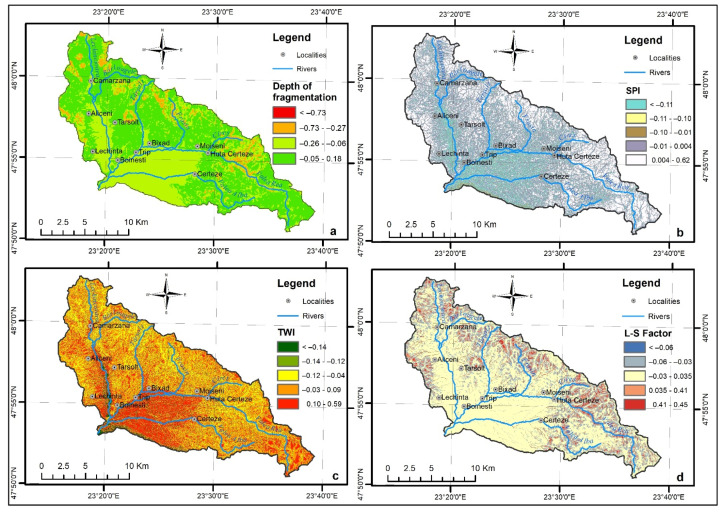
(**a**) Depth of fragmentation, (**b**) Stream Power Index, (**c**) Topographic Wetness Index, (**d**) L-S Factor.

The depth of fragmentation represents a morphometric conditioning factor, which indicates the degree of deepening of the drainage network within a certain catchment area compared to the initial level of the topographic surface, marked by the watershed (Figure 4a). The depth of fragmentation map was computed based on the DEM, applying the cartograms method in the Zonal Statistic tool from the Spatial Analysis extension of ArcMap 10.7.1 software. A fishnet with a cell size of 100 m was created in advance and the differences between the highest and lowest altitudes inside each square were calculated. The manual classification method was used to delineate its values into five classes: 0–2, 2–4, 4–8, 8–16, 16–110. The areas affected by torrentiality are mostly present in the class from 2–4, representing 52.7% of the total torrential pixels, thus, representing the only class with a positive WofE (0.18). This factor received a weight of 8% in the final FFPI equation (Table 1).

### 4.6. The Stream Power Index

The Stream Power Index (SPI) (Figure 4b) represents the product of the catchment area and slope and measures the erosive power of flowing water per unit length of the stream [47,48]. Negative values indicate areas with topographic potential for deposition while positive values indicate potentially erosive areas. The lowest values represent relatively flat areas, influencing the susceptibility to floods, sediment deposition, and accumulation. SPI raster was obtained based on parameters derived from DEM. Its values were determined by implementing the Equation (1) in Map Algebra of ArcMap 10.7.1. software.

SPI = “flow_accumulation” × cell_size × Tan(“slope_degree” × 0.017543)(3)

Based on the natural breaks method, SPI values were divided into five classes: (−13.8)–(−11.3), (−11.2)–(−4.3), (−4.2)–(−2.5), (−2.4)–0.52, 0.53–11.4. Positive values classes received the highest WofE value (0.62), indicating that erosion processes are present in riverbeds and torrential formations. This factor received a weight of 2% in the final FFPI map determination (Table 1).

### 4.7. The Topographic Wetness Index

The Topographic Wetness Index (TWI) represents a morphometric conditioning factor. Taking into consideration the topography of the area, the slopes or flat surfaces, TWI indicates the moisture of the soil (Figure 4c). This flow conditioning variable map was computed by using the DEM as input data in the Basic Terrain Analysis of SAGA GIS 7.9.0. software. In order to divide the values into five classes, we used the natural breaks method: 0–2.49, 2.5–6.0, 6.1–8.0, 8.1–11.6, 11.7–30.2. The areas affected by torrentiality are mostly present in the class between 6.1 and 8.0, which represents 40.5% of the study area. The highest WofE value (0.59) was allocated to the class from 11.7–30.2. Having an important influence, this factor received a weight of 8% on the FFPI map computation (Table 1).

### 4.8. L-S Factor

The L-S factor is another morphometric conditioning factor, commonly used to assess the compound influence of slope length and slope steepness on the occurrence of surface runoff [49,50]. The L-S was determined based on the spatial analysis Equation (2) proposed by Mitasova et al. 1996 [51] and implemented in Map Algebra of ArcMap 10.7.1. software (Figure 4d).

POW ([accumulation] × 3/22.1, 0.6) × POW(sin([slope] × 0.017)/0.09, 1.3)(4)

where: [accumulation]—runoff accumulation, 3—grid resolution, 0.6, 1.3, 22.1, 0.017—experimental coefficients, [slope]—terrain slope.

The L-S factor values were classified into five classes based on the manual break method: 0–2, 2.1–6, 6.1–10, 10.1–50, 50.1–190. The highest WofE (0.45) corresponds to classes between 10.1 and 50. Same as TWI, it received 8% weight on the FFPI map computation (Table 1).

### 4.9. Topographic Position Index

**Figure 5:**
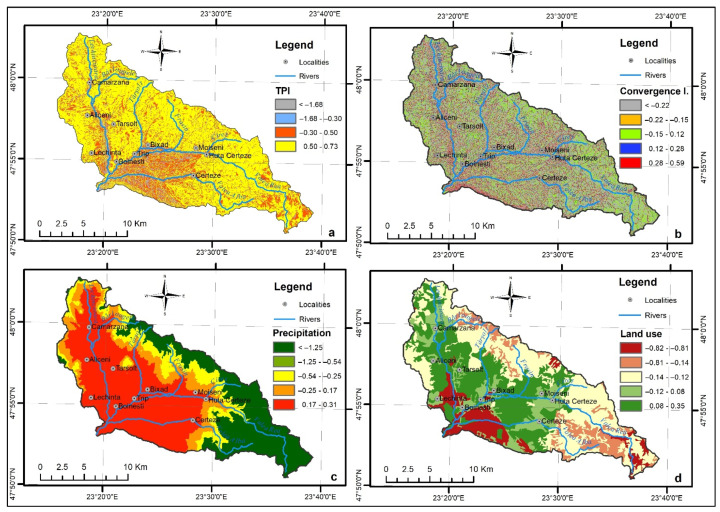
(**a**) Topographic Position Index, (**b**) Convergence Index, (**c)** Precipitation, (**d**) Land use.

The Topographic Position Index (TPI) was also computed by using the DEM as input data in the Basic Terrain Analysis of SAGA GIS 7.9.0. software (Figure 5a). TPI is a scale-dependent flash flood conditioning factor, used to compare the altitude of each cell with the mean altitude of the neighbourhood around that cell [52]. The natural breaks method was used to divide the TPI values as follows: (−35.3)–(−7.10), (−7.09)–(−2.1), (−2.09)–1.66, 1.67–6.98, 6.99–44.5. Positive values represent locations that are higher than the average of their neighbourhood (ridges), negative ones represent locations that are lower than their neighbourhood (valleys), while values around zero are either flat areas or areas with constant slope. In fact, the highest value of the WofE (0.73) was assigned to the class with the lowest value, where the drainage of the surface water is produced. This factor received a 5% weight in the final FFPI map determination (Table 1).

### 4.10. Convergence Index

The Convergence Index (CI) represents a very important flash flood conditioning factor (Figure 5b). By using the aspects of surrounding cells, it calculates an index of convergence regarding overland flow. The negative values of the Convergence Index correspond to convergent areas (valleys), the positive ones to divergent (watershed areas) flow conditions. As in the case of TWI and TPI, the Convergence Index was derived from SAGA GIS 7.9.0. software using the DEM as input data in the Basic Terrain Analysis. The CI factor values were classified into five classes according to the literature [26]: 0.1–99, (−0.9)–0, (−1.9)–(−1), (−2.9)–(−2), (−99)–(−3). The highest WofE (0.59) corresponds to the class between (−99) and (−3), where 30.8% of the total torrential pixels are situated. In the final FFPI equation, it received a weight of 7% (Table 2).

### 4.11. Precipitation

Because of its intensity and spatial distribution, the precipitation represents one of the most essential and important flash flood conditioning factors [42,43]. The multi-annual mean amount of precipitation (MaP) map was derived with the help of Curveexpert by statistically correlating the altitude with the MaP values (Figure 5c). We used the manual classification method to categorise its values into five representative classes: 800–850, 850–900, 950–1000, 1000–1050, 1050–1209. The class between 850 and 900 mm/year covers 32% of the study area and most of the torrential pixels, at approximately 65.8%. As a consequence, the same class received the highest WofE (0.31), followed by the class between 800 and 850, with 0.17 of the WofE. Similar to CI, it received a weight of 7% on the FFPI map computation (Table 2).

### 4.12. Land Use

Due to its influence on the runoff velocity by different coefficients and, thus, on the water balance of a drainage system, land use (Figure 5d) constitutes a deterministic flash flood conditioning factor [51]. It was derived from the Corine Land Cover 2018 dataset, and 12 land use categories were identified in this study area: discontinuous urban fabric; non-irrigated arable land; fruit trees and berry plantations; pastures; complex cultivation patterns; land principally occupied by agriculture, with significant areas of natural vegetation; broad-leaved forest; coniferous forest; mixed forest; natural grasslands; transitional woodland-shrub; sparsely vegetated areas. The broad-level forest category covers most of the study area, representing 35.8% of the catchment area, followed by pastures and land use for fruit trees and berry plantations, which cover 14.7% and 13.6% of the area respectively. These three categories comprise over 75.5% of the total torrential pixels. The highest WofE (0.35) corresponds to the “land principally occupied by agriculture, with significant areas of natural vegetation” category. In the final FFPI equation, land use received the second-highest weight of 10% (Table 2).

### 4.13. Lithology

**Figure 6:**
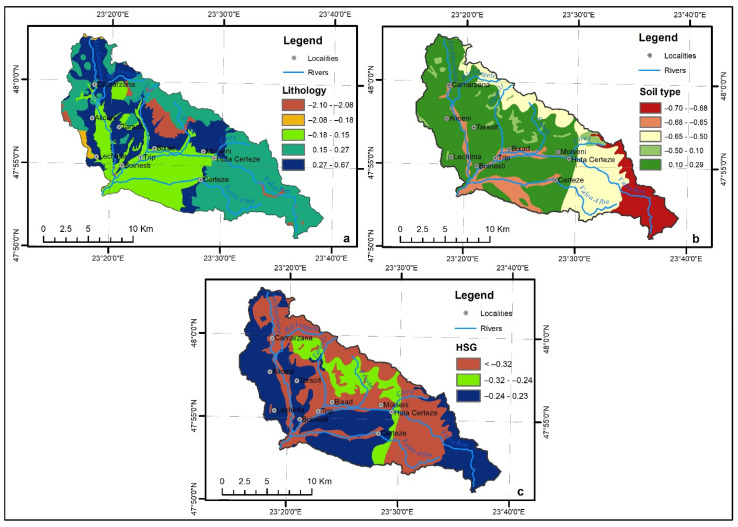
(**a**) Lithology, (**b**) Soil type, (**c**) Hydrologic soil group.

Lithology (Figure 6a) has a vital influence on the surface runoff due to the fact that this factor controls the water infiltration rate. The lithology classes were extracted from the Geological Map of Romania (1:200.000), and 11 categories were identified in the study area. Approximately 36.2% of the study area is covered by basaltic andesites, which represent 20.1% of the total torrential pixels. The second best-represented class is the one containing argillaceous marls/marlstones, sand, and gravel, covering 23.6% of the total area, and including 32.6% of the total torrential pixels. The highest WofE of 0.67 was associated with the category covered by alluvial deposits and proluvium, mostly located at the base of torrential formations. Similar to SPI and elevation, this factor received only a weight of 2% in the final FFPI map computation (Table 2).

### 4.14. Soil Type

The soil type (Figure 6b) represents a basic flash flood conditioning factor, in the context in which water infiltration depends upon soil properties. There are eight soil type classes in the study area, extracted from the Soil Map of Romania (1:200.000). The dominant presence of the broad-leaved forest across the entire study area determines the large extension of several soil types, such as acid brown soils representing 22.4% and brown luvic soils covering 21.8% of the catchment area. The class of brown luvic soils covers 42.3% of the total torrential formation pixels and also received the highest WofE of 0.29. Considered equally important to TPI in the final FFPI equation, the soil type received a 5% weight (Table 2).

### 4.15. Hydrologic Soil Group

The hydrologic soil group (HSG) represents an important geographical factor that reveals the water infiltration rate (Figure 6c). Its definition is based on the association with the soil texture and four hydrologic soil groups were classified, according to [53,54]. In the Valea Rea catchment area, only three hydrologic soil groups were identified, B, C, and D. The dominant soil groups are group D, covering 46.9% of the study area, characterised by a low infiltration rate and a high runoff potential (clayed-loam/clayed texture), and group B, respectively, covering 41.7%. Group B has a medium infiltration rate, a medium runoff potential, and a loamy/loamy-sand texture. It covers 71.3% of the total torrential pixels, receiving the highest WofE of 0.23. Considered as important as the land use factor, HSG received 10% weight on the FFPI map computation (Table 2).

### 4.16. FFPIWofE Distribution in the Valea Rea River Catchment

**Figure 7:**
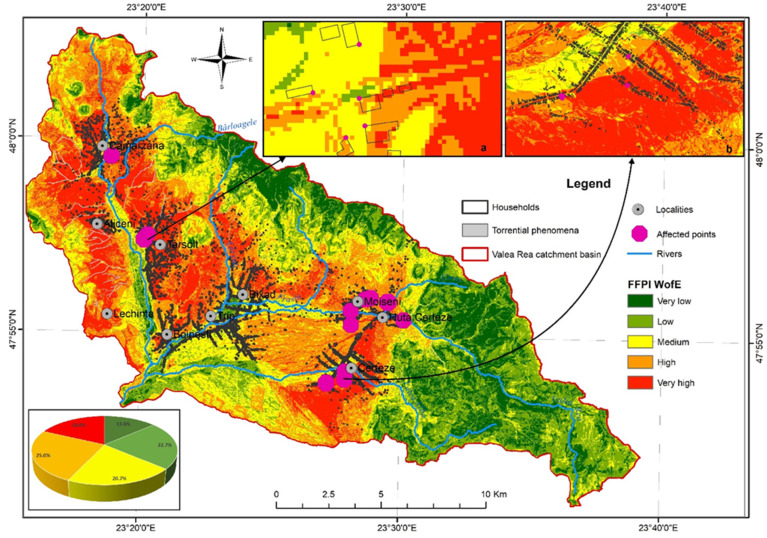
FFPI_WofE_ distribution in the Valea Rea River catchment, (**a**) validation area 1, (**b**) validation area 2.

The integration of the unitarily analysed factors for the acquisition of the final databases, representative for the analysed territory from FFPI perspective, has been carried out according to the weighted average overlay method. The result was a spatial database, classified on five classes (from very low to very high), representing the territorial vulnerability in terms of FFPI (Figure 7).

The analysis at the level of the study area highlights the very large extension of two distinctive categories in terms of territorial vulnerability. First of all, one emphasises the large extension of the low vulnerability class identified in the North, East, and Southeast of the study area, having a weight of approximately 22.7%. This large extension in the above-mentioned areas is caused mainly by the forest vegetation cover of those areas, correlated with a sudden vegetation transit from forests to complex crops in the context of very steep slopes. The low and very low vulnerability classes are also identified across large areas with low slopes in the Lechincioara Valley, Frasin Valley, and Alba Valley, usually located at the confluence of streams and in the small basins created by these valleys. The class of high vulnerability is identified on approximately 25.0% of the territory and is specific especially for the steep slopes of the Lechincioara Valley, the slopes of the lower Târșolț catchment, and the middle areas of the Frasin Valley and Alba Valley catchments. The correlation analysis highlights the fact that the very high vulnerability class is determined by the slope factor, in addition to inadequate land use (orchards and improper crops), as well as lithology and type of soils.

A medium class is identified and may be considered as a transition level between the two extremes previously presented, covering 20.7% of study area. This class is mainly present in the central northern part of the study area, in the southern part, and in the steep transition area, where it is less developed spatially. Analysing the results obtained as a consequence of the implementation of the spatial analysis model on three vulnerability classes: low (low and very low vulnerability), medium (spatially covering the transition area), and high (high and very high vulnerability), the high vulnerability of the study area in terms of FFPI is clearly emphasised because this class spatially covers approximately 43% of the analysed study area.

The territorial impact of the flash flood development potential highlights the risks associated with the territory. This risk may impact the transport infrastructure, the residential areas, as well as the productive agricultural lands located on the steep slopes of the Lechincioara and Târșolț valleys. At the level of the territory, there is major impact due to the fact that the entire class of high vulnerability covers the built-up areas of the villages within the analysed region and is also manifest in the areas with the highest density of residential households and their associated infrastructure.

The validation of the spatial analysis model was performed by directly comparing the obtained results with places where flash floods occurred, randomly identified in the study area. Therefore, a number of 15 sites were identified according to bibliographical references which mentioned the events produced and the damage assessment as a result of the event impact (operational reports, synthetic reports, disaster records). These sites are distributed across the entire study area. For validation, two sites have been selected in order to cover as much of the analysed area as possible. The analysis of the two areas chosen for validation highlights the spatial identification of a number of five points affected by a previous flash flood in areas modelled as having high potential for flash flood occurrence, and one point in an area modelled as having medium potential for flash flood occurrence, within the first area selected for validation (Figure 7a). In the case of the second area selected for validation, a number of four locations were identified as corresponding to an area of high vulnerability, and one location was identified in an area modelled as having very high vulnerability (Figure 7b). The identification of locations affected by previous flash floods in areas corresponding to high and very high vulnerability classes in terms of FFPI during the validation process emphasises the high degree of validation for the proposed model. It also validates the methodological approach and leads to the recommendation that the obtained results should be used and scientifically applied in practice.

## 5. Discussion

The ever-higher intensity and frequency of slope flash floods determines a higher necessity for the drawing up of associated hazard and vulnerability studies, which are increasingly important. Most of the studies are performed at the local level by the authorities responsible for the integrated management of catchment areas, concerning areas already affected by extreme events. These are post-event studies, not taking into account the prediction of such events to mitigate the potential damage. A series of studies were presented at the national level [28,33,34,35] and were validated by means of case studies, proposing methodologies and factors to be considered for FFPI identification. The structure of the presented model fits the general methodologies based on the statistical analysis to identify the territorial vulnerability to slope flash floods, implementing a number of new factors (depth of fragmentation, soil type, precipitation) which influence their development and propagation. The presented model is based on the integration of a number of 15 factors that are materialised in databases acquired directly or based on spatial analysis submodels. The integration of a very large number of databases acquired either from diverse sources or from spatial analysis submodels renders a very high complexity of the proposed model and a very high accuracy of the final result and of its validation. The integration of the 15 factors according to the weighted average equation induces an element of subjectivity within the model when the weight of each factor is established. Therefore, taking into account the mechanisms governing the development of flash floods and based on expert knowledge analysis, it was decided to assign the highest weight to the slope factor, followed by land use and HSG. These factors have an impact on the occurrence of flash floods by reducing the amount of water leading to runoff by interception and infiltration, at the same time influencing the flash flood propagation in terms of direction, speed, erosive power, and impact. The factors that have the lowest weight are represented by lithology, SPI, and elevation, which do not have a direct influence on runoff.

The selected manner of validation for the proposed model (direct validation) suggests its successful use in scientific practice, considering that events once produced in an area have a high probability to occur once again if measures meant to mitigate and reduce them are not taken. In the scientific literature, one may identify a high number of studies using the statistical method of validation, which involves a certain degree of subjectivity, not entirely capturing the territorial specificity. By means of the presented case study, we emphasised both the territorial specificity of flash flood occurrence and development, and the direct correlations between the factors involved in the development of the process. The large coverage of the high class of vulnerability across extensive areas highlights the potential risk associated with the territory. Risk assessment is an absolutely necessary stage in the process of integrated territorial management to highlight viable areas for the development of human activities. Future studies regarding the analysed area will focus on risk assessment concerning all territorial infrastructures and the solutions meant to mitigate these risks.

## 6. Conclusions

Analysing the entire research approach reveals that the proposed methodology may be easily implemented into different study areas. The databases used in the modelling process represent the basis of the spatial analysis model. They are acquired by deriving from DEM and implementing spatial analysis equations. The presented methodology was applied to identify the areas prone to flash floods in different degrees, from very low to very high vulnerability. It also highlights that areas with high and very high vulnerability cover approximately 43% of the total catchment, highlighting the excessively torrential nature of the study area and the potential risks for the territorial infrastructure. Additionally, the identification of large areas of the analysed territory that have high and very high vulnerability underlines the success rate of the model and its validation based on the principle of direct comparison between the result of the model and the reality in the field.

The analysis of the proposed methodology and of the obtained results highlights the following aspects:

- The identification of critical areas where measures should be implemented to mitigate the susceptibility to flash floods by prohibiting massive deforestation and implementing a clear policy of afforestation according to the norms in the field;
- The local and central government should implement policies to prioritise the investments to defend the population from flash floods and to make the inhabitants aware of them. These policies should be based on previous case studies and should underline the negative effects of flash floods;
- The proposed methodology may be also used as a starting point to identify hotspots with high vulnerability and territorial risk within catchment areas that are not supervised by direct measurements;
- The model may be applied on a large scale, reducing the costs related to the implementation of the spatial planning process meant to mitigate the losses inflicted by flash floods. It also reduces the costs for the integrated management of catchment areas.

The databases are easy to obtain and to analyse using proprietary and open-source software, available to any specialist in the field of spatial analysis. The model proposed in this study may represent a methodology to be applied in spatial planning studies, highly useful in land management and for local governments in mitigating the risk of flash floods.

## Figures and Tables

**Table 1:** Flash flood predictor variables classes with their WofE results.

Predictor Variables	Class	Pp (%)	Pt (%)	WofE	WAI (%)
Elevation	145–300	42.6	82.3	0.29	2
	300–450	24.5	15.2	−0.21	
	450–650	17.9	2.4	−0.87	
	650–850	10.2	0.2	−1.80	
	850–1239	4.7	0.0	-	
Slope angle	0–3	22.0	17.2	−0.11	15
	3.1–7	19.8	34.1	0.24	
	7.1–15	32.6	35.6	0.04	
	15.1–25	17.1	10.5	−0.21	
	>25	8.5	2.6	−0.51	
Aspect	Flat/Southwest	17.0	9.4	−0.26	3
	South	13.0	10.7	−0.09	
	Southeast/West	28.7	26.4	−0.04	
	East/Northwest	24.1	24.3	0.00	
	North/Northeast	17.3	29.3	0.23	
Profile curvature	Convex −209–0	50.6	33.9	−0.17	8
	Flat 0–1.92	47.2	55.6	0.07	
	Concave 1.92–199	2.2	10.5	0.69	
Depth of fragmentation	0–2	27.5	22.0	−0.10	8
	2–4	35.1	52.7	0.18	
	4–8	20.3	17.9	−0.05	
	8–16	11.8	6.4	−0.26	
	16–110	5.3	1.0	−0.73	
SPI	(−13.8)–(−11.3)	5.6	4.4	−0.10	2
	(−11.2)–(−4.33)	22.0	17.0	−0.11	
	(−4.32)–(−2.55)	39.3	38.5	−0.01	
	−2.54 –0.52	31.1	31.4	0.00	
	0.53–11.4	2.1	8.7	0.62	
TWI	0–2.49	6.6	4.7	−0.14	8
	2.50–6.04	25.8	19.5	−0.12	
	6.05–8.06	44.1	40.5	−0.04	
	8.07–11.6	21.3	26.7	0.10	
	11.7–30.2	2.2	8.5	0.59	
L-S Factor	0–2	67.1	62.5	−0.03	8
	2–6	23.1	20.4	−0.06	
	6–10	5.9	6.4	0.04	
	10–50	3.7	10.3	0.45	
	50–190	0.1	0.3	0.41	
TPI	(−35.3)–(−7.10)	3.5	18.3	0.73	5
	(−7.09)–(−2.1)	16.5	52.5	0.50	
	(−2.09)–1.66	57.3	28.8	−0.30	
	1.67–6.98	18.6	0.4	−1.68	
	6.99–44.5	4.2	0.0	-	

**Table 2:** Flash flood predictor variables classes with their WofE results.

Predictors Variables	Class	Pp (%)	Pt (%)	WofE	WAI (%)
Convergence Index	0.1–99	53.3	31.9	−0.22	7
	(−0.9)–0	26.6	19.0	−0.15	
	(−1.9)–(−1)	8.3	11.0	0.13	
	(−2.9)–(−2)	3.8	7.3	0.28	
	(−99)–(−3)	7.9	30.8	0.59	
Precipitation	800–850	14.3	21.2	0.17	7
	850–950	32.0	65.8	0.31	
	950–1000	13.6	7.6	−0.25	
	1000–1050	13.1	3.8	−0.54	
	1050–1209	27.1	1.5	−1.25	
Land use	Discontinuous urban fabric	5.7	4.8	−0.12	10
	Non-irrigated arable land	8.0	0.0	-	
	Fruit trees and berry plantations	13.6	26.7	0.25	
	Pastures	14.7	19.6	0.08	
	Complex cultivation patterns	7.2	9.2	0.06	
	Land principally occupied by agriculture, with significant areas of natural vegetation	3.6	9.0	0.35	
	Broad-leaved forest	35.8	29.2	−0.13	
	Coniferous forest	0.3	0.0	-	
	Mixed forest	1.3	0.0	-	
	Natural grasslands	9.3	1.6	−0.82	
	Transitional woodland-shrub	0.5	0.0	-	
	Sparsely vegetated areas	0.1	0.0	-	
Lithology	Amphibole andesites	0.1	0.0	-	2
	Basaltic andesites	36.2	20.1	−0.17	
	Quartz andesites	5.8	2.6	−0.35	
	Pyroclastic rocks	4.3	4.4	0.01	
	Argillaceous marls/marlstones, sand, gravel	23.6	32.6	0.14	
	Andesites	0.6	1.2	0.27	
	Alluvial deposits, proluvium	3.2	14.8	0.67	
	Porphyry granodiorites	0.6	0.9	0.19	
	Diluvium	11.1	0.1	−2.10	
	Gravel, sand, and argillaceous sand	13.4	19.2	0.16	
	Porphyry diorite	1.0	4.1	0.59	
Soil type	Acid brown soils	22.4	7.1	−0.50	5
	Brown luvic (podzolic) soils	21.8	42.3	0.29	
	Clayish brown luvisols	15.0	22.9	0.18	
	Lithosols	3.4	2.6	−0.12	
	Albeluvisols (podzoluvisols)	18.5	23.1	0.10	
	Eu-mesobasic brown soils	9.2	2.0	−0.70	
	Andosols	9.7	0.0	-	
	Alluvial soils	0.0	0.0	-	
HSG	D	46.9	22.3	−0.32	10
	B	41.7	71.3	0.23	
	C	11.4	6.4	−0.25	

Pp (%)—percentage of class pixels; Pt (%)—percentage of torrential pixels; WAI—Weighted Average Integration.

## Data Availability

Not applicable.
